# Exploring the evolving function of soluble intercellular adhesion molecule-1 in junction dynamics during spermatogenesis

**DOI:** 10.3389/fendo.2023.1281812

**Published:** 2024-01-08

**Authors:** Xiang Xiao, Yating Han, Qin Li, Dongwang Zheng, C. Yan Cheng, Ya Ni

**Affiliations:** ^1^ Center for Reproductive Health, School of Pharmaceutical Sciences, Hangzhou Medical College (Zhejiang Academy of Medical Sciences), Hangzhou, China; ^2^ Engineering Research Center of Novel Vaccine of Zhejiang Province, Hangzhou Medical College, Hangzhou, China; ^3^ School of Basic Medical Sciences and Forensic Medicine, Hangzhou Medical College, Hangzhou, China; ^4^ School of Pharmaceutical Sciences, Hangzhou Medical College, Hangzhou, China; ^5^ Department of Urology and Andrology, Sir Run-Run Shaw Hospital, Zhejiang University School of Medicine, Hangzhou, China

**Keywords:** soluble intercellular adhesion molecule-1 (sICAM-1), Sertoli cells, blood-testis barrier (BTB), testis, spermatogenesis, cytoskeleton, male fertility

## Abstract

Intercellular adhesion molecule-1 (ICAM-1) is a transmembrane glycoprotein expressed on immune, endothelial, and epithelial cells. Its ectodomain can be proteolytically cleaved to release a circulating soluble form called sICAM-1. Clinical studies demonstrate sICAM-1 is upregulated in various diseases and associated with disease severity. Research has identified sICAM-1 as a regulator of the blood-testis barrier (BTB) and spermatogenesis. Overexpression of sICAM-1 weakened the BTB *in vitro* and *in vivo*, downregulated junction proteins including N-cadherin, γ-catenin, and connexin 43, and caused germ cell loss. This contrasts with barrier-strengthening effects of membrane-bound ICAM-1. sICAM-1 may act as a molecular switch enabling germ cells to open BTB and Sertoli-germ cell adhesion for transport across the seminiferous epithelium. While the mechanism remains unclear, reduced SRC family kinase (SFK) signaling was observed following sICAM-1 overexpression. SRC promotes BTB protein endocytosis and degradation, influences cytoskeletal dynamics, and affects cell polarity. As sICAM-1 overexpression phenocopies SRC inhibition, SRC may operate downstream of sICAM-1 in regulating BTB dynamics and spermatogenesis. Investigating sICAM-1’s structure-function regions and downstream targets will elucidate the molecular mechanisms of junction disruption. This knowledge could enable strategies targeting sICAM-1/SRC to modulate BTB permeability and treat male infertility or diseases involving endothelial/epithelial barrier dysfunction.

## 1 Introduction

Male infertility is a major global health challenge according to a recent World Health Organization (WHO) report analyzing infertility prevalence worldwide from 1990 to 2021. The report found that around 17.5% of adults, approximately 1 in 6 people, experience infertility, with comparable rates across high-, middle-, and low-income countries. Male infertility negatively impacts the physical, psychological, and social well-being of men of reproductive age worldwide. A separate study estimated that over 56 million men suffered from infertility up to 2019, representing a 76.9% increase from 1990 (1). In addition to impacts on reproductive capacity, male infertility also causes substantial psychosocial distress and introduces treatment costs (2, 3).

Multiple factors can contribute to male infertility, including abnormalities in sperm function and quality, as well as failure to produce sperm. However, the precise molecular mechanisms remain poorly understood, making diagnosis and treatment challenging. Improved semen parameters after treatment do not guarantee identifying the underlying causes (4–8). Studies show close links between male fertility and overall health. Infertility increases risks of illnesses unrelated to reproduction, such as cancer, diabetes, and cardiovascular disease. Infertile men also face higher hospitalization and mortality rates when seriously ill (9–11). This correlation suggests a coordinated regulatory system involving male reproduction and other body systems. Shared effector molecules and regulatory proteins may underlie different disease processes.

One such molecule is intercellular adhesion molecule-1 (ICAM-1), which exists in both membrane-bound and circulating soluble forms (sICAM-1). In rodent testes, germ cells appear to secrete sICAM-1 to alter Sertoli cell adhesion as they move across the seminiferous epithelium during spermatogenesis. Overexpression of sICAM-1 in rat testes severely impairs the blood-testis barrier (BTB) function and germ cell adhesion (12–14). Clinical evidence has associated elevated sICAM-1 levels with diverse pathological states, including male infertility (**Table 1**). This positions sICAM-1 as a putative shared effector that links reproductive and systemic damage. However, the mechanism behind sICAM-1’s action remains unclear. A comprehensive investigation into sICAM-1’s impacts on testis function and on Sertoli and germ cell adhesion is needed. Deeper insights into sICAM-1’s role and mechanisms could provide therapeutic targets for infertility while elucidating systemic disease mechanisms. This could significantly advance infertility prevention and treatment, as well as therapies targeting common regulatory pathways for systemic diseases.

**Table 1:** sICAM-1/ICAM-1 expression in body fluids across different pathological conditions^a^.

Diseases		Body fluid(s)	sICAM-1	ICAM-1	Pathway(s)	Inhibitor	Ref(s)
Autoimmune diseases	Celiac disease	Serum	↑				(15)
	Graves’ ophthalmopathy	Serum	↑			Carbimazole	(16–18)
	Idiopathic pulmonary fibrosis	Serum	↑	↑			(19)
	Psoriasis	Serum, plasma	↑		IL-18↑		(20–23)
	Rheumatoid arthritis	Serum, plasma, synovial fluid	↑		TNF-α↑	TNFi	(24–26)
	Scleroderma	Serum	↑				(27)
	Spontaneous urticaria	Serum	↑				(28)
	Systemic lupus erythematosus	Plasma	↑	↑			(29, 30)
	Ulcerative colitis	Serum	↑			Prednisolone	(31)
	Vitiligo	Skin tissue fluid	↑	↓	IL-6↑, IL-17↑, TNF-α↑		(32, 33)
Cancers	Colorectal cancer	Serum	↑		TNF-α↑		(34–37)
	Gastric cancer	Serum	↑	↑			(38–40)
	Invasive bladder cancer	Urine, serum	↑	↑			(41)
	Lung cancer	Serum	↑	↑	TNF-α↑		(42–45)
	Mammary cancer	Serum	↑				(46, 47)
Cardiovascular diseases	Coronary heart disease	Blood	↑		IL-6↑, sTNFR1↑, sTNFR2↑		(48)
	Dilated cardiomyopathy	Serum	↑				(49)
	Hypertension	Plasma	↑				(50)
Liver Diseases	Alcoholic liver cirrhosis	Serum	↑	↑	TNF-α↑		(51)
	Cholestasis	Serum	↑				(52)
	Chronic hepatitis	Serum	↑			IFN-α	(53)
	Primary biliary cirrhosis	Serum	↑	↑			(54)
	Primary sclerosing cholangitis	Serum	↑	↑			(54)
	Schistosomiasis japonica	Serum	↑				(55)
Nervous system diseases	Alzheimer’s disease	Cerebrospinal fluid	↑		IL-8 ↑		(56, 57)
	Aseptic meningitis	Cerebrospinal fluid	↓		IL-8 ↑		(58)
	Bipolar disorder	Blood	↑	↑			(59)
	Schizophrenia	Plasma	↑			chlorpromazine	(59)
	Multiple sclerosis	Serum, cerebrospinal fluid	↑	↑			(60–63)
Other diseases	Endometriosis	Peritoneal fluid	↑				(64)
	Thoracic inflammation	Pleural fluid	↑				(65)
	Male infertility	Seminal plasma	↑		IL-6↑		(66)

a This table provides an illustrative, non-exhaustive summary of sICAM-1/ICAM-1 levels in various pathological states. Carbimazole, TNFi, prednisolone, IFN-α and chlorpromazine have been shown to decrease sICAM-1 levels and are therefore considered inhibitors. Abbreviations used in this table include: IL (Interleukin), TNF (Tumor Necrosis Factor), sTNFR1 (Soluble Tumor Necrosis Factor Receptor-1), sTNFR2 (Soluble Tumor Necrosis Factor Receptor-2), IFN-α (Interferon-α), and TNFi (Tumor Necrosis Factor Inhibitor). Up-regulation is indicated by an upward arrow (↑) and downregulation is indicated by a downward arrow (↓).

## 2 Background of sICAM-1

### 2.1 Origins and variants

ICAM-1, also known as Cluster of Differentiation 54 (CD54), is a single-chain transmembrane glycoprotein that consists of an extracellular region with five immunoglobulin (Ig)-like domains, a transmembrane segment, and a short cytoplasmic tail. ICAM-1 is expressed at relatively low levels by immune cells, endothelial cells, epithelial cells, and other normal tissues. However, multiple inflammatory stimuli, including cytokines such as tumor necrosis factor (TNF)-α, interleukin (IL)-1, and interferon (IFN)-γ, as well as lipopolysaccharide (LPS), can increase ICAM-1 expression through transcription (67–70).

**Figure 1:**
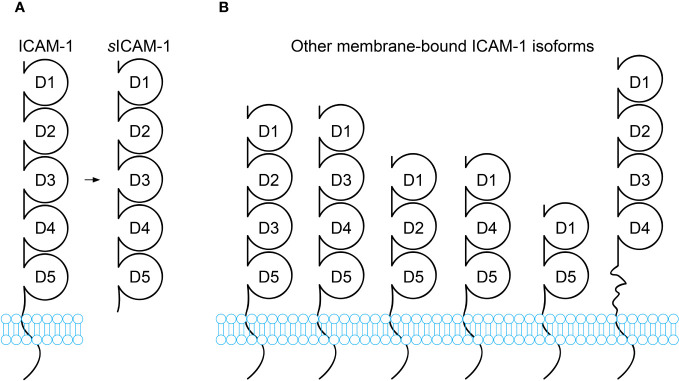
Schematic diagram of ICAM-1 isoforms. **(A)** Full-length ICAM-1 is a transmembrane protein containing 5 immunoglobulin(Ig)-like extracellular domains (D1-D5), a transmembrane domain, and a short cytoplasmic domain. Soluble ICAM-1 (sICAM-1) lacks the transmembrane and cytoplasmic domains. **(B)** In addition to the full-length isoform, alternative splicing generates transmembrane ICAM-1 isoforms with truncated extracellular regions containing 2, 3, or 4 Ig-like domains. While these alternatively spliced isoforms have been reported, most functional research pertains to full-length ICAM-1. The isoforms likely differ in tissue distribution, expression levels, and functions. Some may only appear under certain conditions, such as pathological conditions.

Under inflammatory or cellular stress conditions, the ectodomain of ICAM-1 can be proteolytically cleaved and shed from the cell surface. This releases the soluble form, sICAM-1, into extracellular fluids. Pro-inflammatory cytokines enhance this shedding, leading to increased sICAM-1 levels. Circulating in body fluids like blood, sICAM-1 is a truncated form of ICAM-1 that consists solely of the five Ig-like extracellular domains D1-D5, without the transmembrane and cytoplasmic regions (**Figure 1A**). Being heavily glycosylated, the molecular weights of ICAM-1 and sICAM-1 can vary between 75-115 kDa and 50-90 kDa respectively (13, 70–72).

The origin and generation of sICAM-1 is not fully understood. It is thought to primarily occur through the proteolytic cleavage of ICAM-1’s ectodomain by proteases. Proteases like serine proteases, matrix metalloproteinases (MMPs), and members of the “a disintegrin and metalloproteinase” (ADAM) family may cleave at different sites on ICAM-1. Both the ICAM-1 cleavage by different proteases and the differences in glycosylation could produce variants of sICAM-1 with subtle structural differences. Alternative mRNA splicing can also generate other variants of sICAM-1. Six other splice variants of ICAM-1 have been reported, varying in their combination and number of Ig domains (**Figure 1B**) (73). These variants are more susceptible to proteolytic cleavage and may contribute additional sICAM-1. Some studies suggest mRNAs that encode sICAM-1 directly may exist (13, 74). Each of these mechanisms could give rise to sICAM-1 variants that differ in structure and function. Studies using isoform-deficient mice found they have sharply contrasting disease phenotypes. The ability of ICAM-1 variants to bind ligands like lymphocyte function-associated antigen-1 (LFA-1) *in vitro* also varies depending on present Ig domains. Expression of isoforms differs between cell types and may change with inflammation (73). However, it remains unclear if these isoforms could differentially regulate ligand interactions, dimerization, intracellular signaling and disease outcomes through their structural variations expressed on different cell types. It is still unknown if any hypothetical fragments have unique roles. Nevertheless, the only soluble form found in body fluids contains domains D1-D5, appearing most physiologically relevant (13, 70, 74). Further work is needed to characterize and compare sICAM-1 variants from splicing versus protease cleavage to understand their properties and potential functions. This may provide insight into the complex origins of sICAM-1.

### 2.2 General functions and disease associations

sICAM-1 plays complex roles in regulating inflammation and immunity. It is proposed that sICAM-1 retains the characteristics of membrane-bound ICAM-1 and can compete with ICAM-1 for binding to the integrin receptor LFA-1. As a competitive inhibitor, sICAM-1 can influence leukocyte adhesion and migration by inhibiting ICAM-1-mediated interactions between leukocytes and endothelial cells. This potentially weakens the body’s immunity (70, 74, 75). The concentration of sICAM-1 affects cytokine release, immune cell adhesion, angiogenesis and tissue repair in different ways. At low concentrations, sICAM-1 can promote cytokine release and immune cell activation. In contrast, high levels of sICAM-1 may limit leukocyte adhesion while promoting angiogenesis and tissue repair (70). Specific genetic variations in the ICAM-1 gene can also affect sICAM-1 levels (69). Additionally, sICAM-1 acts as a signaling molecule. The intensity of signaling induced by sICAM-1 is regulated by the completeness of N-glycosylation and sialylation. Sialylation does not affect ICAM-1 binding to LFA-1 in astrocytes. However, it is critical for the signaling function of sICAM-1 in inducing production of the inflammatory chemokine MIP-2/CXCL2 (macrophage inflammatory protein 2/C-X-C motif chemokine ligand 2). Fully sialylated sICAM-1 induces a more rapid, robust, and prolonged MIP-2 response compared to nonsialylated or high mannose glycoforms. Sialylation may regulate receptor interactions and signaling kinetics of sICAM-1 (76).

sICAM-1 has been detected in various human body fluids including serum, cerebrospinal fluid, bile, amniotic fluid, and urine (71, 72, 74, 77–80). Elevated sICAM-1 levels correlate with disease severity and prognosis in numerous diseases like cancer, cardiovascular disease, autoimmune disorders, nervous system disease, inflammation, and viral infections (70, 72, 80–86). For instance, recent studies have found that higher levels of sICAM-1 in the serum of COVID-19 patients are positively associated with disease severity and can even predict the risk of mortality (81, 82). In chronic pain patients, serum sICAM-1 levels have been found to significantly correlate with pain intensity. However, in acute experimental pain models with healthy volunteers, sICAM-1 levels did not directly correlate with perceived pain levels. Instead, sICAM-1 underwent short-term changes after acute nociceptive stimuli (87). Additionally, an increase in sICAM-1 levels in seminal plasma may be associated with immune infertility in men (66).

Unlike ICAM-1 whose cellular expression is difficult to clinically assess, sICAM-1 in body fluid is easily measurable and is often used as a common marker for inflammatory diseases (71, 74, 77–79). However, the mechanism behind its action is still unclear. Efforts to therapeutically modulate sICAM-1 levels have shown limited efficacy in treating diseases so far. Currently, most research focuses on exploring relationships between sICAM-1 levels in fluids and disease onset, progression, and prognosis (75, 82, 84, 88) (**Table 1**). Alternatively, studies have generally viewed ICAM-1 and sICAM-1 as ubiquitous adhesion molecules expressed on epithelial or endothelial cell surfaces in response to environmental stimuli. It is believed that high levels of sICAM-1 may stem from corresponding vascular endothelial dysfunction (70, 72). For example, research has demonstrated that high sICAM-1 levels in cerebrospinal fluid are associated with increased phosphorylation of the microtubule-associated protein tau, which is implicated in blood-brain barrier (BBB) dysfunction, and with elevated levels of total tau protein. However, few studies have examined the links between sICAM-1 and endothelial barrier permeability. Limited existing findings suggest that sICAM-1 can influence the transport of immune cells across the BBB and their communication with surrounding cells (72, 89–92).

The precise impact of sICAM-1 on germ cell transport across the BTB and throughout the seminiferous epithelium remains unclear. It also remains unknown whether sICAM-1 acts as a key signaling molecule for communication between germ cells and surrounding Sertoli cells. At present, these mechanisms remain mysterious, as only one study to date has linked sICAM-1 to spermatogenesis and BTB permeability (12). In the upcoming section of this review, cell adhesion and junction dynamics in the testis will first be introduced. Hypothetical molecular mechanisms for the role of sICAM-1 during spermatogenesis will then be proposed. Exploring potential explanations could help further the understanding of how sICAM-1 may regulate both the BTB and germ cell adhesion. Ultimately, this may provide useful insights to advance future research on sICAM-1’s function. While questions remain, continued investigation of sICAM-1’s involvement holds promise to elucidate the intricate process of spermatogenesis.

## 3 Cell adhesion and junction dynamics in the testis

### 3.1 Cell junctions facilitate cell adhesion

Cell migration and morphogenesis, which are central to developmental processes, require dynamic changes in cell adhesion properties. Fundamentally, cell adhesion refers to interactions between cells (cell-cell adhesion) and between cells and the extracellular matrix (ECM, cell-ECM adhesion). These interactions play a pivotal role in maintaining tissue integrity, homeostasis, and function. Cell adhesion is primarily facilitated through specialized structures known as cell junctions, which include tight junctions (TJ), adherens junctions (AJ), desmosomes, and gap junctions (GJ). These junctions work in concert to provide structural stability, coordinate cellular behavior, and maintain tissue architecture. Nonetheless, each junction type serves unique and indispensable roles in connecting cells, facilitating intercellular signaling, and providing cell adhesion. TJ, composed of integral membrane proteins such as occludin and claudins, interact with the actin cytoskeleton through adapter proteins like ZO-1, thereby forming a permeability barrier through tight sealing between cells. This restricts the passage of molecules and plays an integral part during the development and remodeling of epithelial tissue. AJ, with core components like cadherins/catenins and nectins/afadin complexes, typically form first between cells during epithelial development, providing mechanical attachment. This is followed by formation of TJ at the apical region to AJ, sealing the paracellular space. At desmosomes, the adhesion proteins desmoglein and desmocollin provide linkage to intermediate filaments within cells. GJ, formed by clustering of connexins, create intercellular channels that directly transfer molecules between cells (93–97).

### 3.2 Specialized junctions in the testis

**Figure 2:**
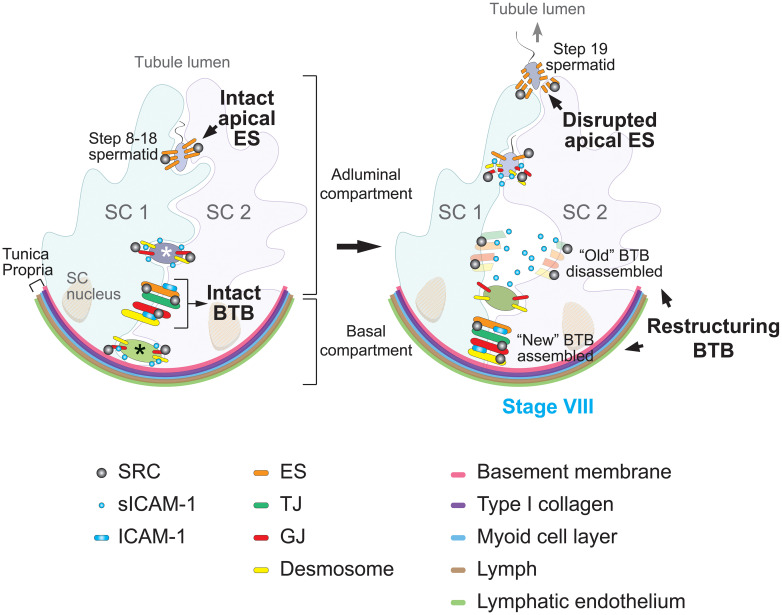
Proposed model of sICAM-1 and SRC signaling in regulating blood-testis barrier (BTB) dynamics and spermatogenesis. In the rat seminiferous tubule (left), the seminiferous epithelium is composed of Sertoli cells (SC) and germ cells at different developmental stages. The BTB, formed between adjacent Sertoli cells (SC 1 and SC 2), divides the epithelium into basal and adluminal compartments. Less mature germ cells (such as spermatogonia and preleptotene spermatocytes, marked with a black asterisk) reside in the basal compartment, while more advanced germ cells (such as primary and secondary spermatocytes, as well as round spermatids, marked with a white asterisk) reside in the adluminal compartment. The BTB comprises four distinct cell junction types, color-coded in the diagram: basal ectoplasmic specializations (ES), tight junctions (TJ), gap junctions (GJ), and desmosomes. Sertoli cells also form apical ES with step 8 and beyond spermatids, acting as the primary anchoring junction until spermiation. Pre-step 8 germ cells are linked to Sertoli cells through GJ and desmosomes. Src functions as a pivotal regulator of both the BTB and Sertoli-germ cell adhesion. At stage VIII of the seminiferous epithelial cycle (right), the BTB restructures to enable transit of preleptotene spermatocytes. This includes the formation of a “new” BTB beneath spermatocytes and the disintegration of the “old” BTB above the spermatocytes, enabling spermatocyte passage into the adluminal compartment without compromising the BTB. Concurrently, the apical ES is broken down to release mature sperm, specifically the step 19 spermatid. During these events, SRC facilitates BTB remodeling and regulates the dynamics of Sertoli-germ cell junctions, particularly apical ES disintegration. Through protein endocytosis and degradation pathways, SRC promotes transport of spermatocytes across the BTB and release of mature spermatozoa from the seminiferous epithelium. Previous studies show membrane-bound ICAM-1 promotes BTB assembly, whereas its soluble form sICAM-1 expressed by germ cells impairs the BTB to facilitate spermatocyte transit. Overexpression of sICAM-1 also disrupts GJ and desmosomes between Sertoli and germ cells, causing loss of immature germ cells. SRC may act downstream of sICAM-1 to regulate BTB restructuring and Sertoli-germ cell adhesion during spermatogenesis.

In the adult mammalian testis, spermatogenesis exemplifies the vital role of cell adhesion and junction dynamics. This highly coordinated and cyclical process involves the continual division and differentiation of germ cells, which are tightly bound to the surrounding Sertoli cells within seminiferous tubules. Cell-cell adhesion is mediated by TJ, ectoplasmic specializations (ES), GJ, and desmosomes, all relying on Sertoli cells (98). ES are unique testis-specific actin-based AJ structure located at basal sites between adjacent Sertoli cells. Characterized by their hexagonal actin arrays sandwiched between the Sertoli cell plasma membrane and endoplasmic reticulum, basal ES comprise part of the BTB. They coexist and intermix with the other three junction types (TJ, GJ, and desmosomes) to form the intricate, multifaceted BTB structure (99–102), as depicted in **Figure 2** showing the different junction types labeled with colors.

ES are also found at apical sites between Sertoli cells and spermatids, termed apical ES. Once formed, apical ES serve as the sole anchoring junction at these sites until spermiation. Different Sertoli-germ cell junction types predominate during specific maturation stages, enabling diverse germ cell activities (98–105). In rodent models, early germ cells including spermatogonial stem cells, spermatogonia, spermatocytes, and pre-step 8 spermatids, primarily connect to Sertoli cells through GJ and desmosomes. However, as germ cells mature into step 8 and beyond spermatids, apical ES takes precedence (99–101, 103, 104). This junctional shift underscores the complexity and specificity of cell-cell interactions within the dynamic environment of the testis. At apical ES sites, hexagonal actin filament bundles are restricted to the Sertoli cell side. Apical ES anchor spermatids and guide their orientation and bidirectional movement in the epithelium, potentially via linkage to microtubule motors like kinesins and dyneins (106–110).

### 3.3 BTB dynamics

The BTB is a defining feature of the testis, fundamentally consisting of specialized cell junction structures that create a selectively permeable barrier within the seminiferous epithelium. It separates the epithelium into adluminal (apical) and basal compartments (**Figure 2**) and constitutes one of the tightest tissue barriers in the body. The BTB prevents substances in the blood like drugs and antibodies from accessing the adluminal compartment, providing an immunological and physical shield to developing germ cells. However, the BTB must be dynamic—continuous reorganization of intricate junctional complexes enables spermatocyte transit from the basal to adluminal compartment, a critical step in sperm development. This meticulous BTB remodeling facilitates extensive germ cell transport while maintaining barrier integrity (102).

The BTB’s constant restructuring without compromising its immunological barrier function is made possible by coordinated interplay between its cell junction components. As shown in **Figure 2**, movement of preleptotene spermatocytes across the BTB involves localized assembly of “new” junctions below transiting spermatocytes, followed by disassembly of “old” junctions above them. Accordingly, junctional proteins undergo endocytosis, then intracellular transport including protein degradation, recycling back to the cell surface, and transcytosis across cells (102, 105, 111–113). This intricately orchestrated BTB dynamics enables the extensive germ cell development needed for the remarkable sperm production capacity of the mammalian testes.

## 4 Detection and roles of sICAM-1 in the rat testis

### 4.1 Expression of sICAM-1

A previous study has shown that both Sertoli cells and germ cells express membrane-bound ICAM-1 in the rat testis, as examined using commercial antibodies against the cytoplasmic region of ICAM-1 (12). During stage VIII of the seminiferous epithelial cycle, ICAM-1 expression at the BTB significantly increases when examined by immunofluorescence microscopy, coinciding with BTB restructuring and the transit of preleptotene spermatocytes across it as they differentiate into leptotene and zygotene spermatocytes. This suggests ICAM-1 involvement in spermatocyte transport across the BTB. However, these antibodies cannot recognize sICAM-1. Using a custom polyclonal antibody targeting the extracellular D2-D3 domain of ICAM-1, the authors detected sICAM-1 in rat testes. The identified sICAM-1 comprised all five Ig-like domains and had a molecular weight of around 70 kDa. This is lower than the full-length ICAM-1 in rat testes with a molecular weight of approximately 97 kDa. The antibody also detected additional protein fragments with lower molecular weights, indicating potential alternative forms of sICAM-1 may be present (12). By immunoblotting analysis, sICAM-1 was found to be highly expressed in germ cells (12). This suggests germ cells may secrete sICAM-1 to regulate Sertoli cell adhesion and facilitate their own crossing of the BTB and transport in the epithelium (12–14).

### 4.2 Contrasting roles of sICAM-1 and ICAM-1

The authors further discovered that overexpression of sICAM-1, via a plasmid containing only the extracellular domains D1-D5, exerted an opposing effect on BTB permeability compared to overexpression of the full-length membrane-bound ICAM-1 (12). While overexpression of full-length ICAM-1 strengthens the BTB, overexpression of sICAM-1 severely impairs BTB function and causes loss of adhesion between spermatocytes and round spermatids with supportive Sertoli cells within the seminiferous epithelium (12–14).

Specifically, overexpression studies in a Sertoli cell culture model, which mimics the BTB *in vitro*, found ICAM-1 and sICAM-1 have antagonistic effects on Sertoli cell barrier function (12). ICAM-1 strengthened the barrier, mimicking “new” BTB assembly, whereas sICAM-1 weakened it, corresponding to “old” BTB disassembly during restructuring (**Figure 2**). Moreover, overexpressed sICAM-1 downregulated expression of BTB proteins including N-cadherin (an ES protein), γ-catenin (the only known protein present at both ES and desmosomes, also called plakoglobin (114–116)), and connexin 43 (a GJ protein). These differential effects were verified *in vivo*, where sICAM-1 overexpression in rat testes disrupted the BTB, downregulated N-cadherin and connexin 43, and caused loss of spermatocytes and round spermatids (step 1-7 spermatids). Compared to membrane-bound ICAM-1, sICAM-1 may act as a molecular switch and promoter of germ cell transit across the BTB. By interfering with Sertoli-germ cell junctions and BTB protein expression, sICAM-1 may facilitate transport of germ cells during differentiation.

In summary, sICAM-1 dually assists germ cell movement by disrupting adhesion at ES, desmosomes and GJ, and downregulating proteins like N-cadherin, γ-catenin and connexin 43 (**Figure 2**). This contrasts with membrane ICAM-1 which reinforces adhesion. sICAM-1 thus fine-tunes BTB dynamics to support spermatogenesis.

## 5 The mechanisms of sICAM-1 regulation of cell junctions

### 5.1 sICAM-1 downregulates SRC signaling pathways

The mechanisms by which sICAM-1 downregulates the expression of BTB and cell adhesion proteins, or “opens” cell junctions in the testis remain unclear. Overexpression studies in cultured primary Sertoli cells provide insights. sICAM-1 overexpression also reduced levels of signaling molecules important in SRC signaling, including SRC, PYK2, p-SRC-Y530 and p-PYK2-Y402 (12). This implies SRC pathways likely mediate intracellular sICAM-1 effects. Phosphorylation at tyrosine 530 (Y530) of SRC renders the kinase in an inactive confirmation (117). Thus, reduced p-SRC-Y530, in the context of decreased total SRC, indicates lower inactive and overall SRC levels, suggesting either a general decline in SRC activity, or a shift in the normal balance between active and inactive SRC conformations. As a known SRC substrate, PYK2 autophosphorylation at tyrosine 402 (Y402) recruits SRC to further regulate PYK2 activity (118). Diminished SRC and p-PYK2-Y402 levels indicate potential downregulation of PYK2 signaling involved in cell adhesion, migration, and possibly calcium-induced signaling events (119, 120). However, more research on PYK2 in the testis is needed. Collectively, this data shows sICAM-1 overexpression decreases SRC-related signaling pathways. This supports SRC pathways as probable intracellular mediators of sICAM-1.

### 5.2 Role of SRC in regulating BTB dynamics

SRC family kinases (SFKs), a family of non-receptor tyrosine kinases, play crucial roles in various cellular processes through signal transduction. Key members involved in the testis include SRC and YES. SFKs are known to regulate cell adhesion by modulating adhesion complexes and cytoskeletal rearrangement. They also participate in junction remodeling by phosphorylating junction proteins. Additionally, SFKs regulate endocytosis through phosphorylating endocytic vesicle proteins (121–124).

Previous studies have shown SFKs, particularly SRC, are critical modulators of BTB dynamics and spermatogenesis. SRC alters the phosphorylation state of BTB and apical ES proteins. This triggers endocytosis and intracellular transport of the junctional components, controlling opening/closing of the BTB and dissociation of spermatozoa from Sertoli cells via disruption of the apical ES (98, 102, 105). Specifically, during stage VIII of the seminiferous epithelial cycle in rodent testes, BTB restructuring coincides with sperm release through apical ES disruption (**Figure 2**). SRC promotes BTB disintegration and apical ES disruption by inducing protein endocytosis and degradation, mediating both “old” BTB disassembly during spermatocyte transit and loss of mature sperm association with Sertoli cells during spermiation. SRC also facilitates adhesion between immature germ cells and Sertoli cells. In summary, SRC signaling plays multifaceted regulatory roles on BTB and Sertoli-germ cell interactions throughout spermatogenesis (124–129). While its precise interaction with sICAM-1 requires further clarification, they may coordinately influence critical junction events.

### 5.3 Similar phenotypes suggest SRC mediates sICAM-1 effects

Previous research shows that SRC contributes to the organization and remodeling of F-actin structures in Sertoli cells (124, 128). Excess sICAM-1 undermines SRC signaling in rat testes, severely impacting the structure and alignment of actin filaments (F-actin) (12), corresponding to SRC knockdown phenotypes (128). In other words, overexpressing sICAM-1 or inhibiting SRC produce similar results on Sertoli cell F-actin cytoskeleton.

Additionally, SRC is known to regulate intracellular protein transport, facilitating junction dynamics and aiding “old” BTB disassembly during spermatocyte transit (124–128). It binds and interacts with specific BTB protein complexes, such as N-cadherin/β-/γ-catenin, desmoglein-2/γ-catenin, and connexin 43/plakophilin-2 (102, 124, 130). SRC phosphorylation of N-cadherin, β-catenin, or γ-catenin leads to catenin dissociation from N-cadherin at the BTB sites, resulting in cadherin/catenin complex degradation, breakdown of basal ES at the BTB, and consequently increasing BTB permeability (13, 102, 130–132). These SRC-induced effects closely resemble effects of overexpressing sICAM-1, which downregulates BTB proteins like the N-cadherin/γ-catenin complex, impairing overall BTB integrity.

Excess sICAM-1 in rat testes also led to a disordered arrangement of post-step 8 spermatids within the seminiferous tubules. The sperm heads, rather than maintaining a neat alignment towards the basement membrane, exhibited a loss in polarity. The SRC-PYK2 pathway is associated with cell polarity and is likely involved in germ cell transport in the seminiferous epithelium (126, 133, 134). As sICAM-1 overexpression reduced the protein levels of SRC and PYK2, it may impede the SRC-PYK2 signaling in Sertoli cells, potentially impairing germ cell polarity and their movement within the seminiferous epithelium. Evidence also shows that intraperitoneal injection of the SRC inhibitor PP1 into rats induces sloughing of spermatocytes and round spermatids from the seminiferous epithelium (130). This phenotype of germ cell loss mimics the effects of excessive sICAM-1 in the testis. Together, sICAM-1 overexpression reduces SRC-related signaling while phenocopying global SRC inhibition outcomes, such as disoriented spermatids and their premature leaving. Thus, when secreted by germ cells, sICAM-1 appears to target SRC in Sertoli cells, affecting Sertoli-germ cell adhesion.

### 5.4 Summary and future directions

**Figure 3:**
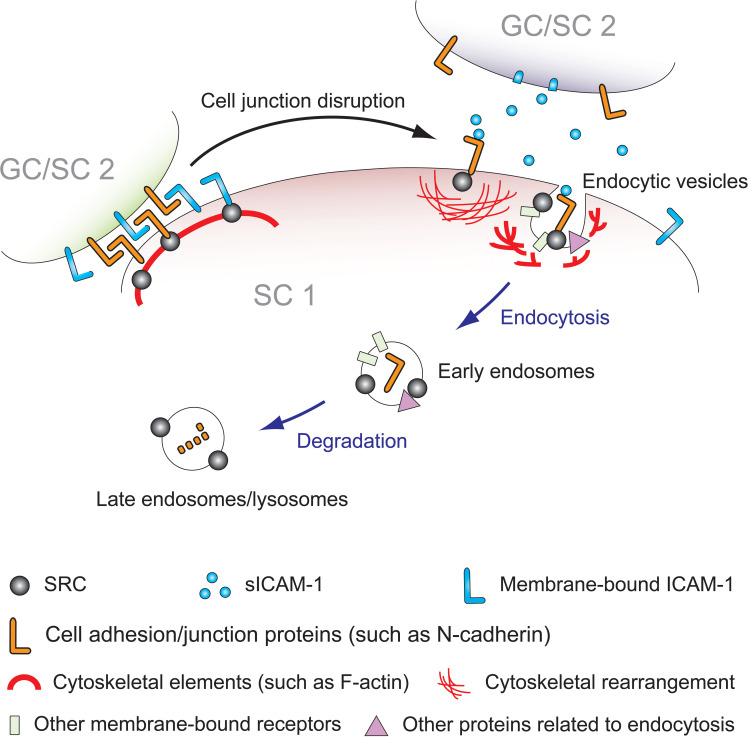
Schematic model depicting the mechanism of sICAM-1 and SRC interplay during Sertoli-Sertoli (SC 1 and SC 2) and Sertoli-germ (SC 1 and GC) cell adhesion. In mammalian testes, germ cell adhesion and the dynamic reorganization of the blood-testis barrier (BTB) primarily rely on protein endocytosis and intracellular transport of internalized proteins, including protein degradation, recycling, and transcytosis. SRC is known to regulate adhesion between immature germ cells and Sertoli cells, as well as the release of mature sperm from the seminiferous epithelium, playing a role in cell junction dissociation and restructuring. Previous studies have shown that SRC promotes endocytosis and degradation of BTB proteins in Sertoli cells, disassembling the “old” BTB. Overexpression of sICAM-1 in primary Sertoli cells and rat testes damages the BTB, GJ, and desmosomes, leading to increased BTB permeability, loss of immature germ cells, and decreased expression levels of SRC, p-SRC-Y530, and SRC substrate PYK2/p-PYK2-Y402. This affects the F-actin cytoskeleton, consistent with the phenotype of SRC deficiency or inhibition. As sICAM-1 is a germ cell-secreted extracellular signal, SRC likely acts downstream as the intracellular mediator of sICAM-1 effects on Sertoli cells, propagating sICAM-1 disruption of the BTB and Sertoli-germ cell adhesion. This model illustrates that at Sertoli-Sertoli (BTB) and Sertoli-germ cell junctions, sICAM-1 signals through cell adhesion proteins to recruit SRC, triggering cytoskeletal reorganization and adhesion protein endocytosis and degradation to regulate the BTB and spermatogenesis.

In conclusion, the evidence presented here demonstrates that sICAM-1 and SRC both contribute to junction disassembly and F-actin cytoskeleton remodeling. sICAM-1 overexpression diminishes activity of the tyrosine phosphorylation pathways driven by SRC and its related kinases. This impairs GJ and/or desmosomes, leading to BTB dysfunction and premature germ cell release. This pattern mirrors the effects seen when overall SRC activity is suppressed. Regarding F-actin cytoskeleton, the consequences of sICAM-1 overexpression parallel those of an SRC deficiency. Evidence also indicates sICAM-1 overexpression reduces levels of SRC and PYK2, possibly inhibiting downstream signaling in Sertoli cells, which could impair germ cell polarity and transport by disrupting SRC-PYK2. SRC thus mediates sICAM-1 impacts on BTB integrity, Sertoli-germ cell adhesion, and underlying cytoskeleton. When germ cells secrete sICAM-1 at the Sertoli-Sertoli or Sertoli-germ cell interface, SRC in Sertoli cells responds, so as to facilitate germ cell transport via cytoskeletal modulation and/or junction restructuring (**Figure 3**).

Future directions could explore these proposed mechanisms further. Research has indicated that the four predominant cytoskeletal structures in the testis—namely, microfilaments (F-actin), microtubules (MT, composed of tubulin polymers), intermediate filaments (e.g., vimentin filaments), and septins—interact with and influence each other (135–137). Additional investigations may delve into the impacts of sICAM-1 overexpression on these various cytoskeletal structures, as well as the subsequent changes in the Sertoli cell cytoskeleton after modulating SRC expression or activity. Approaches that alter SRC might offer a way to counteract the adverse outcomes instigated by sICAM-1 overproduction, such as BTB compromise, premature germ cell loss, and defects in spermatid polarity.

Further studies could also investigate transcription factors and broader gene expression changes resulting from sICAM-1-mediated effects on SRC signaling. The testis has specialized transcription complexes that coordinate the spermatogenic differentiation program (138). SRC can phosphorylate and/or activate various transcription factors like forkhead box class O proteins (FOXO), signal transducer and activator of transcription (STAT) proteins, and nuclear factor-κB (NF-κB) (122, 139–141). Low sICAM-1 levels may also trigger NF-κB and ERK activation, releasing inflammatory cytokines (70). However, limited information exists on specific testicular transcription factors regulated by SRC or the sICAM-1/SRC pathway. It would be interesting to decipher how main transcriptional factors are regulated in response to changes in phosphorylation status of the SRC kinase signaling pathway, and also understand the potential effect of signaling mediated by sICAM-1 on the transcriptional networks that are activated or downregulated in response to changes in sICAM-1/ICAM-1 ratios during spermatogenesis. Profiling genome-wide expression changes at defined spermatogenic stages after sICAM-1 exposure could reveal roles of particular transcription factors and gene networks altered by SRC-mediated signals. Integrating transcriptomic and proteomic data will enable constructing detailed signaling cascades from surface sICAM-1/ICAM-1 ratios and adhesive interactions to nuclear transcriptional responses governing spermatogenesis and cytoskeletal remodeling.

In summary, sICAM-1 and SRC are pivotal regulators of BTB function and spermatogenesis. Overproduction of sICAM-1 mirrors the effects of suppressing SRC, implying that SRC may be positioned downstream of sICAM-1, governing the Sertoli cell cytoskeleton, BTB stability, and the adhesion of germ cells. A more in-depth understanding of the sICAM-1/SRC signaling mechanisms promises to yield valuable insights for manipulating BTB permeability and addressing male infertility.

## 6 Understanding sICAM-1 interactions and knowledge gaps

### 6.1 sICAM-1 interactions and binding partners

ICAM-1 and its soluble form sICAM-1 must interact with various partners to mediate their functions. One of the best characterized binding partners is LFA-1, a member of the β2-integrin family. As the classical interacting protein of ICAM-1, studies show that sICAM-1 can competitively bind to LFA-1 similarly to ICAM-1 (142, 143). However, the precise role of this interaction in the testis remains unclear. While LFA-1 has been detected in mouse testicular germ cells, it does not appear to directly bind ICAM-1 in this tissue (144). This suggests there may be alternative, testis-specific binding partners for both ICAM-1 and sICAM-1 that influence their function locally. Co-immunoprecipitation experiments shows ICAM-1 physically associates with actin cytoskeletal filaments as well as several important tight junction proteins in the rat testis, including occludin and N-cadherin (12). These proteins represent plausible interacting partners for ICAM-1 and sICAM-1 at testicular junctional sites. However, it remains unknown whether the associations are direct or indirect. Further studies are still needed to conclusively identify the ligand(s) that ICAM-1 and sICAM-1 bind to on adjacent Sertoli and germ cells.

Questions also remain regarding the precise mechanisms and functional outcomes of these putative interactions in the testicular environment. Do ICAM-1 and sICAM-1 directly engage occludin, N-cadherin or other proteins at junctions? Elucidating their binding modes of action could provide crucial insights into how these molecules regulate testicular permeability and germ cell transport.

### 6.2 Functional regions of sICAM-1

Beyond its binding partners, understanding the specific domains that facilitate interactions is important for elucidating ICAM-1 and sICAM-1 function. Examining their signaling mechanisms further illuminates the roles of these molecules in the testis.

Structural analyses indicate the D1 domain of ICAM-1 predominantly mediates its binding to transmembrane partners like LFA-1. Additionally, the D3 or D3-D4 regions interact with other β2-integrin family members such as Mac-1 and p150,95 (74). As sICAM-1 likely binds extracellular targets on adjacent Sertoli cells, one hypothesis is that sICAM-1 engages N-cadherin extracellularly and recruits SRC signaling through N-cadherin’s cytoplasmic tail, thereby initiating SRC signaling intracellularly. Analyzing individual or combined sICAM-1 fragments could help identify key motifs enabling these interactions. Understanding the specific domains involved in partner binding and signaling initiation provides insights into the mechanisms by which ICAM-1 and sICAM-1 exert their functions in the testis.

### 6.3 Differences between membrane-bound ICAM-1 and tailless sICAM-1 signaling

The cytoplasmic tail of ICAM-1 plays a pivotal regulatory role in intracellular signaling, influencing various cellular processes (70). Deletion of this tail impairs ICAM-1 function, reducing adhesion and stress fiber formation. The RKIKK motif within the tail plays a critical part in regulating ICAM-1 dynamics on the cell surface, inducing actin cytoskeleton rearrangement and stress fiber formation through interactions with actin binding proteins (145). Additionally, the tail establishes important interactions with the actin cytoskeleton. It is essential for efficient RhoA activation and also interacts with myosin-II and Rac1, contributing to downstream effects on actin cytoskeleton remodeling and cell adhesion (70, 146, 147). Moreover, it is crucial for the ICAM-1 cleavage process, particularly through tyrosine residues Y474 and Y485 within its cytoplasmic region (148). The tail’s association with proteins like α-actinin, ezrin, and moesin is also pivotal for ICAM-1 localization and functions related to adhesion and migration (149, 150).

In contrast to membrane-bound ICAM-1, as the tailless form, sICAM-1 lacks the ability to directly impact key intracellular signaling entities. For example, sICAM-1 cannot influence SRC or components of the actin cytoskeleton in the same way as ICAM-1. The precise mechanisms of sICAM-1 signaling remain uncertain. It is unclear whether signaling solely involves sICAM-1 functioning as a cleaved and released molecule, or if signaling may still occur through residual interactions between the tail and intracellular proteins after cleavage. Alternatively, sICAM-1 could signal independently upon binding extracellular receptors. Elucidating these uncertainties surrounding sICAM-1 signaling pathways requires further investigation.

### 6.4 Target molecules of sICAM-1

While sICAM-1 shares similarities with ICAM-1, emerging evidence indicates it functions as a distinct signaling molecule in the testis (12–14). Both Sertoli and germ cells express ICAM-1, but germ cells predominantly express sICAM-1 and not ICAM-1. Since sICAM-1 overexpression alone triggers downstream signaling, it likely transmits signals generated by germ cells to Sertoli cells. One hypothesis is that sICAM-1 functions to open the BTB, enabling spermatocyte transit across. It may also help interrupt and reassemble Sertoli-germ cell adhesion to facilitate transport of developing germ cells through the epithelium. Elevated sICAM-1 associates with decreased SRC signaling and lower levels of specific adhesion proteins such as N-cadherin, connexin 43, and γ-catenin. Thus, sICAM-1 could prompt degradation of BTB/adhesion proteins like N-cadherin via SRC pathways, compromising junction integrity. N-cadherin and other membrane proteins may be direct targets on Sertoli cells that enter SRC-mediated degradation pathways upon receiving extracellular sICAM-1 signals from germ cells (**Figure 3**). Identifying sICAM-1’s exact molecular targets and deciphering its role in regulating barrier permeability and germ cell movement are fundamental goals for advancing our understanding.

### 6.5 Linking sICAM-1 to testicular pathology and dysfunction

While studies establish associations between sICAM-1 and barrier integrity proteins, several questions remain regarding its links to pathological conditions in the testis. The inverse relationship between sICAM-1 levels and proteins involved in cell contacts points to its potential involvement in adhesion dysregulation. However, causality has yet to be proven. Exposure to toxicants is known to disrupt the BTB and cause germ cell loss. Given sICAM-1’s role in barrier function, it may mediate some aspects of toxicant susceptibility in the testis. Some studies have shown that air pollutants such as diesel exhaust particles and particulate matter up-regulate sICAM-1 and/or ICAM-1 expression in both humans and animals (151–153). Additionally, drug treatments like statins mostly decreased sICAM-1 and/or ICAM-1 levels in patients (154, 155). However, direct evidence in the testis is still lacking. The precise circumstances influencing sICAM-1 level changes in the testis remain unknown, as does the identification of treatments that could reduce its levels.

To move from correlation to elucidating disease mechanisms, more in-depth investigation of sICAM-1 signaling pathways is needed. Answering questions about how it specifically modifies junction formation and barrier properties, as well as its effects in toxicant exposure models, could help link sICAM-1 functions to pathological barrier breakdown in the testis. Addressing key gaps through techniques like overexpression, knockdown and toxicological models will help determine sICAM-1’s precise role in testis dysfunction. Its intricate regulation of cell adhesions indicates its potential as a target for diagnosing and treating male reproductive conditions.

## 7 Future avenues and opportunities for sICAM-1 study

The study of sICAM-1, an endogenous regulatory molecule with unique biological activity, presents promising opportunities to advance our understanding of processes beyond its established role in BTB restructuring and spermatogenesis. One exciting area of future research is the BBB. Like the BTB, the BBB’s vital protective function also prevents drug delivery to the brain. sICAM-1’s ability to modulate barrier function makes it a candidate for strategies to enable efficient drug delivery across the BBB, potentially enabling new treatments for neurological disorders. Pairing sICAM-1 fragments with specific drugs could be explored, developing targeted brain delivery approaches. Additionally, sICAM-1’s association with inflammatory diseases like rheumatoid arthritis suggests the possibility of elucidating molecular mechanisms to inform targeted therapies. Its influence on tumor progression and metastasis also indicates significance for cancer research, perhaps leading to innovative therapeutic approaches for patients. Deeper investigation into sICAM-1’s role across tissue systems may provide pivotal insights. For cases of unexplained male infertility or testicular inflammation, exploring BTB damage and the expression of sICAM-1, SRC, and related signals could illuminate underlying causes. In summary, sICAM-1’s diverse implications across biology and medicine offer promising opportunities to advance both scientific understanding and therapeutic innovation.

## Author contributions

XX: Conceptualization, Funding acquisition, Resources, Supervision, Writing – original draft, Writing – review & editing. YH: Investigation, Methodology, Writing – review & editing. QL: Investigation, Methodology, Writing – review & editing. DZ: Investigation, Methodology, Writing – review & editing. CC: Methodology, Resources, Validation, Writing – review & editing. YN: Conceptualization, Funding acquisition, Methodology, Resources, Supervision, Writing – review & editing.
